# Folding drives cortical thickness variations

**DOI:** 10.1140/epjst/e2020-000001-6

**Published:** 2020-11-16

**Authors:** Maria A. Holland, Silvia Budday, Gang Li, Dinggang Shen, Alain Goriely, Ellen Kuhl

**Affiliations:** 1 Department of Aerospace and Mechanical Engineering, University of Notre Dame, Notre Dame, IN 46556, USA; 2 Department of Mechanical Engineering, Friedrich-Alexander University, 91058 Erlangen, Germany; 3 Department of Radiology and Biomedical Research Imaging Center, University of North Carolina at Chapel Hill, Chapel Hill, NC 27599, USA; 4 Mathematical Institute, University of Oxford, Oxford, UK; 5 Department of Mechanical Engineering, Stanford University, Stanford, CA 94305, USA

## Abstract

The cortical thickness is a characteristic biomarker for a wide variety of neurological disorders. While the structural organization of the cerebral cortex is tightly regulated and evolutionarily preserved, its thickness varies widely between 1.5 and 4.5 mm across the healthy adult human brain. It remains unclear whether these thickness variations are a cause or consequence of cortical development. Recent studies suggest that cortical thickness variations are primarily a result of genetic effects. Previous studies showed that a simple homogeneous bilayered system with a growing layer on an elastic substrate undergoes a unique symmetry breaking into a spatially heterogeneous system with discrete gyri and sulci. Here, we expand on that work to explore the evolution of cortical thickness variations over time to support our finding that cortical pattern formation and thickness variations can be explained – at least in part – by the physical forces that emerge during cortical folding. Strikingly, as growth progresses, the developing gyri universally thicken and the sulci thin, even in the complete absence of regional information. Using magnetic resonance images, we demonstrate that these naturally emerging thickness variations agree with the cortical folding pattern in *n* = 9 healthy adult human brains, in *n* = 564 healthy human brains ages 7–64, and in *n* = 73 infant brains scanned at birth, and at ages one and two. Additionally, we show that cortical organoids develop similar patterns throughout their growth. Our results suggest that genetic, geometric, and physical events during brain development are closely interrelated. Understanding regional and temporal variations in cortical thickness can provide insight into the evolution and causative factors of neurological disorders, inform the diagnosis of neurological conditions, and assess the efficacy of treatment options.

## 1 Motivation

A central question of developmental biology is to understand the interplay of genetic, biochemical, geometric, and physical factors during morphogenesis, the biological development of shape. Understanding the relative role of these factors in the developing human brain is a particularly interesting but also challenging question. The fundamental paradigm that physical forces play a central role during brain development was first proposed more than a century ago by the Swiss anatomist Wilhelm His who postulated that *Entwicklungsmechanik*, developmental mechanics, is the key driver for the characteristic folding pattern of our brain [1]. Interestingly, these ideas have not received much attention during the 20th century aside from the work of LeGros Clark [2] and Hutchinson [3]. Throughout the last few years, however, there has been a flurry of activities in this area [4–10], and our common understanding has now converged to the notion that the folding pattern of our brain is closely correlated to physical forces and morphoelastic instabilities [11–16].

### 1.1 Genetics and biochemistry can explain why the brain folds

A forty-year-old hypothesis [3] suggests that the complex surface morphology of our brain is the natural result of differential growth between the different cortical layers and regions [17]. Recent studies have investigated a similar hypothesis, in which differential growth between gray and white matter leads to gyrification [6,18–21]. This notion is supported by a recent large-scale transcriptomic analysis of the individual germ layers in regions of prospective folds and fissures that found discrete domains of gene expression in the developing gyrencephalic cortex of ferrets, but not in the lissencephalic cortex of mice [22]. More recently, human brain organoids have advanced as a promising model system to study the interplay of genetics, biochemistry, and physics during pattern formation in the developing human brain [23]. Many genes that regulate progenitor proliferation, neurogenesis, and fate specification including key signaling pathways such as Notch, Shh, MAPK, and Wnt, which directly regulate cortical growth, seem to be differentially expressed between future gyri and sulci [24]. This close correlation between structural differentiation and the formation of gyri and sulci agrees with recent in utero magnetic resonance measurements in rhesus macaque fetuses [25]. These observations raise the questions how these discrete regions emerge [26] and what regulates pattern formation [27].

### 1.2 Geometry and physics can explain how the brain folds

**Fig. 1.**
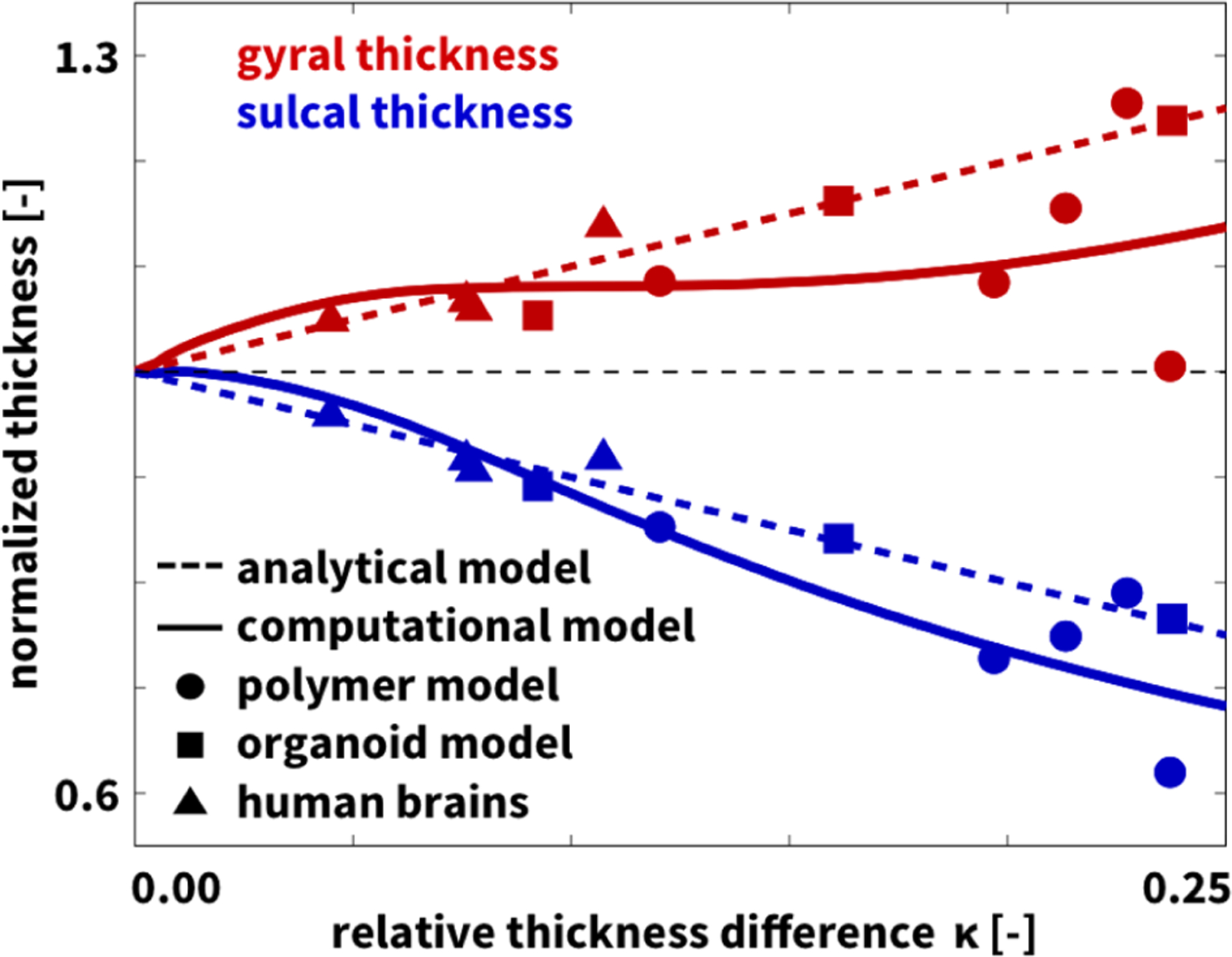
Thickness variations upon folding. Gyri (red) are universally thicker than sulci (blue). Analytical model (dashed lines), computational model (solid lines), polymer model (circles), and organoid model (squares) agree with human brain analysis (triangles). Gyral and sulci thicknesses are normalized by the thickness at the onset of the instability and shown as functions of the relative thickness difference. Figure modified from [20].

The importance of geometry and physics in pattern selection becomes apparent when comparing the cortical folding patterns brains of different shapes [28]: round brains fold more uniformly while longitudinal folds dominate on elongated brains [29]. Cortical thickness is also sensitive to small perturbations during cortical development, and small alterations in early development could result in the large-scale changes seen in neurodevelopmental disorders like lissencephaly and polymicrogyria, but also with autism spectrum disorders [30], schizophrenia [31], and epilepsy [32]. Cortical thickness, calculated as the distance between the pial surface and the gray-white interface [33], changes very little with brain size and is relatively well preserved both within and across different species [34]. Cortical thickness variations in the human brain follow small-world principles [35] and vary between 1.5 and 4.5 mm [36]. Yet, it remains unclear whether variations in cortical thickness are a cause or consequence of cortical folding. To address this question, we expand on our previous work [20] with additional polymer experiments and organoid models and show that folding universally induces a symmetry breaking into a spatially heterogeneous system with thickening gyri and thinning sulci, even in the complete absence of regional information. Figure 1 summarizes the findings of our study in a thickness variation graph.

## 2 Materials and methods

### 2.1 Analytical model

To analytically study thickness variations upon folding, we consider the idealized model problem of a bilayered system at the onset of folding [37] using the method described in [21]. We model the formation of gyri and sulci within the framework of morphoelasticity [15] and consider the morphoelastic instability problem caused by the homogeneous growth of an elastic layer on an elastic substrate [38]. We decompose the deformation gradient, the spatial gradient of the nonlinear deformation map, ***F*** = ***F***^e^ ·***F***^g^, into an elastic contribution ***F***^e^ and a growth contribution ***F***^g^ [39]. We model both layer and substrate as incompressible neo-Hookean materials with a Cauchy stress ***σ*** = *µ* (***F***^e^)^t^ ·***F***^e^–*p* ***I***, where *µ* is the shear modulus, ***I*** is the second order unit tensor, and *p* is the Langrange multiplier that enforces incompressibility. We assume that the layer grows isotropically and morphogenetically, ***F***^g^ = *ϑ* ***I***, parameterized in terms of the growth factor *ϑ*, which we assume to increase gradually at a constant rate, $\dot{\vartheta }=G$. To compute the critical growth factor *ϑ*^crit^ at which the folding instability occurs, we use a variational method that probes the stability of the homogeneous layer by studying emerging folding modes with the lowest elastic energy [40]. This method also characterizes the shape of the folds and the deformations of the layer for values of the growth factor *ϑ* larger than, but close to *ϑ*^crit^. Initially, the layer covers the domain (Y,X)∈[0,1]×ℝ and the substrate the domain (Y,X)∈(−∞,0]×ℝ. We approximate the shape of the folding mode to first order as *y*(*x*, *Y*) = *ϑ*^2^*Y* + *AkF*_1_(*Y*) cos(*kx*), where *A* is the amplitude of the fold, *Y* is the height of a material point in the initial configuration, and growth is *ϑ* = *ϑ*^crit^ in the layer and *ϑ* = 1 in the substrate [20]. We can then determine the thicknesses of the gyri and sulci, normalized by the thickness at the onset of folding, *t*_g_ = [*y*(*π/k*_c_, 1) – *y*(*π/k*_c_, 0)]/(*ϑ*^crit^)^2^ and *t*_s_ = [*y*(0, 1) – *y*(0, 0)]/(*ϑ*^crit^)^2^. In the linear approximation, the thicknesses become *t*_g_ = 1 + *aA* and *t*_s_ = 1 – *aA*, and we can introduce the relative thickness difference *κ* = [*t*_g_ – *t*_s_]*/*[*t*_g_ + *t*_s_] = *aA*. The coefficient *a >* 0 depends on the stiffness ratio *β* = *µ*_l_/*µ*_s_, the ratio between the stiffnesses of layer and substrate, and is positive for all stiffness ratios beyond the folding threshold of *β >* 0.544 [20]. For small stiffness ratios [41], the folding instability becomes a supercritical pitchfork bifurcation [42] and the coefficient *a* decreases as *β* increases. We thus expect the folding pattern to be stable at the onset and conclude that, close to the bifurcation, *gyri increase* in thickness while *sulci decrease*. Figure 1 illustrates our analytical thickness estimates as dashed red and blue lines.

### 2.2 Computational model

To computationally study thickness variations upon folding, we simulate a bilayered model system of a growing stiff thin layer on top of a soft thick substrate [37]. Using the nonlinear field theories of mechanics supplemented by the theory of finite growth [43], we characterize the bilayered system through a set of five equations that define kinematics, constitutive behavior, mechanical equilibrium, growth kinematics, and growth kinetics [18]. Kinematically, we decompose the deformation gradient ***F*** = ***F***^e^ ·***F***^g^ into an elastic contribution ***F***^e^ and a growth contribution ***F***^g^. Similarly, we decompose the overall volume change *J* = det(***F***) = *J*^e^*J*^g^ into an elastic volume change *J*^e^ = det(***F***^e^) and a growth-induced volume change *J*^g^ = det(***F***^g^). Constitutively, only the elastic contributions ***F***^e^ and *J*^e^ generate stresses. We assume a neo-Hookean material behavior with a Cauchy stress, ***σ*** = [λ ln(*J*^e^)–*µ*] ***I***+*µ* (***F***^e^)^t^ ·***F***^e^, where λ and *µ* are the elastic Lamé constants and ***I*** is the second order unit tensor. The stresses enter the mechanical equilibrium equation, div(***σ***) ≐ **0**, which we solve numerically using a custom-designed nonlinear finite element program [44]. To close the set of governing equations, we constitutively prescribe the evolution of growth. We assume that growth is isotropic and purely morphogenetic [4], i.e., independent of physical forces [38], ***F***^g^ = *ϑ* ***I*** where *ϑ* is the growth factor that defines the grown volume $J^{\text{g}}=\vartheta^{n_{\text{dim}}}$ in terms of the number of spatial dimensions *n*_dim_. We postulate that the layer grows linearly $\dot{\vartheta }=G$ at a constant rate *G >* 0 and that the substrate is purely elastic and does not grow, *ϑ* = 1 and *G* = 0 [11]. We create a finite element model of a rectangular subsection of a human brain slice with a normalized width of 11.0 cm, height of 2.5 cm, and initial layer thickness of 0.125 cm, discretized by 240, 20, and 4 elements each. This results in a discretization with 5760 elements and 12 050 degrees of freedom. We assume a plane strain state and fixed the left, bottom, and right boundary nodes orthogonal to the boundary, but allowed them to slide freely along the edge. Motivated by the stiffness ratios in human brain tissue [45], we assume that the layer with Lamé constants of *µ*_l_ = 0.30 kPa and λ_l_ = 2.79 kPa is approximately three times stiffer than the substrate with *µ*_s_ = 0.10 kPa and λ_s_ = 0.93 kPa. We solve the resulting nonlinear finite element equations using a standard Newton-Raphson method [44]. Figure 1 illustrates our computational thickness variations as solid red and blue lines.

### 2.3 Polymer model

To experimentally study thickness variations upon folding, we create a bilayered model system of room-temperature-vulcanization silicone rubber with a stiff thin layer on top of a prestretched soft thick substrate [46]. To cast the soft substrate, we mix a platinum-catalyzed silicone, Ecoflex 00–30 (Smooth-On, Macungie, PA), dye the mixture in white, pour it into a rectangular casting mold, and cure it for 5 h at room temperature. After curing, we uniaxially prestretch the substrate from its initial length *L* to a prestretched length *l* using a custom-built stretching device. To prepare the stiff layer, we mix a platinum silicone, Mold Star 20T (Smooth-On, Macungie, PA), dye the mixture in blue, pour it onto the stretched substrate, and cure it for 2 h at room temperature. To induce folding in the layer, we gradually release the prestretch. We take images of the folded bilayer and characterize the gyral and sulcal thicknesses using digital imaging techniques. We apply a constant prestretch of λ = *l/L* = 2.0 and systematically vary the initial layer thicknesses from 0.30 mm, 0.45 mm, 0.60 mm, 0.80 mm, and 1.00 mm to 2.00 mm. At this prestretch level, layer and substrate have stiffnesses of 324 kPa and 69 kPa, which generates a stiffness contrast of 4.7, about twice the stiffness contrast in the adult human brain. Figure 1 illustrates our gyral and sulcal polymer thicknesses as red and blue circles.

### 2.4 Organoid model

**Fig. 2.**
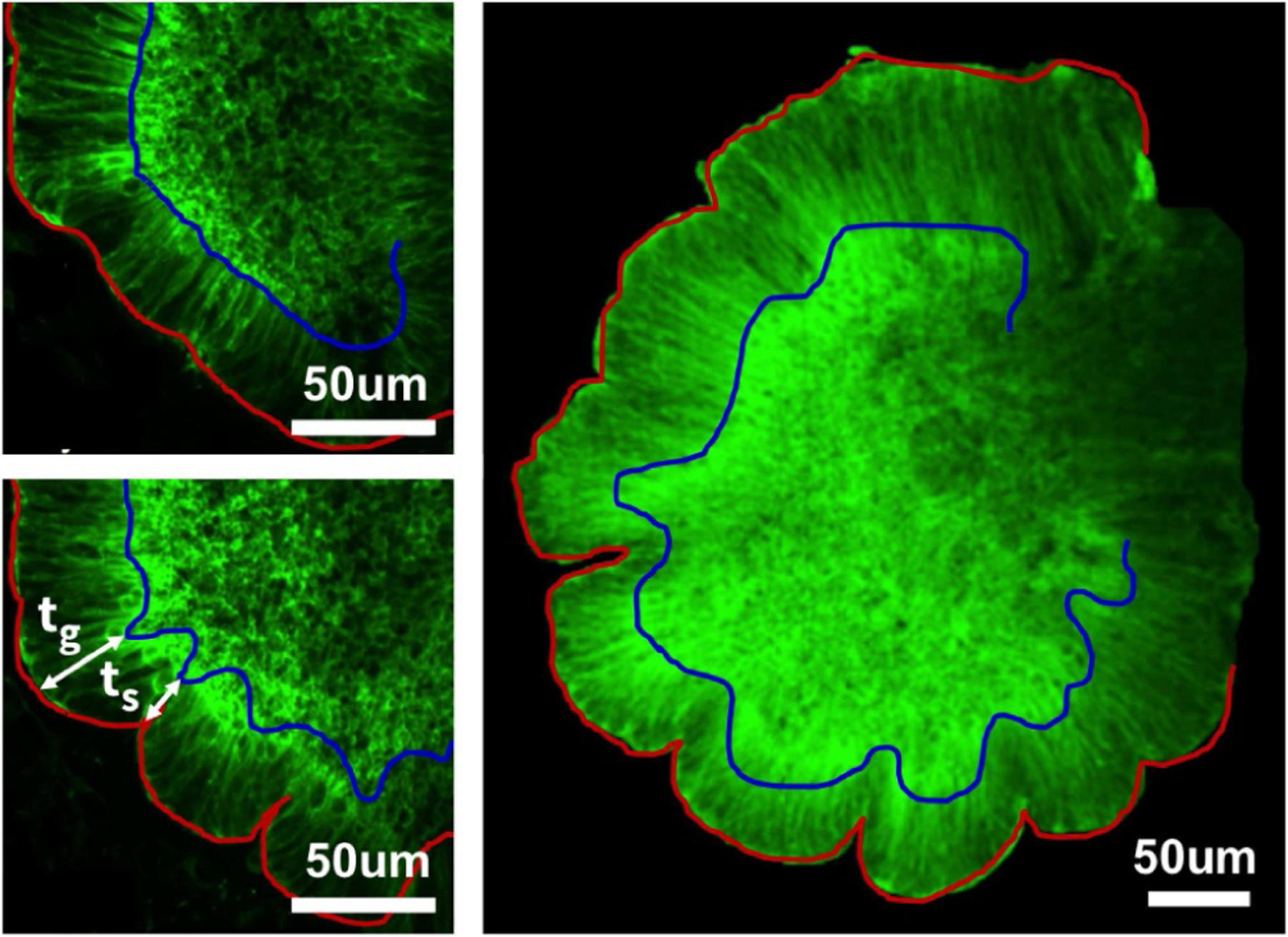
Organoid model. Fluorescence images of human brain organoids at days 6 (top left) and 7 (bottom left) reveal the onset of folding in a living system, adopted with permission from [16]. Reconstructed outer (red) and inner (blue) surfaces (right) illustrate thickening gyri and thinning sulci.

To study thickness variations upon folding in living systems, we analyze fluorescent images of human brain organoids at the onset of folding and beyond. We consult a recent study on human brain organoids on a chip [16] and analyze their fluorescence images of individual organoids. We outline the outer and inner surfaces and characterize the gyral and sulcal thicknesses using digital imaging techniques. Figure 2 shows three representative organoids with the outer and inner surfaces outlined in red and blue. Figure 1 illustrates our gyral and sulcal organoid thicknesses as red and blue squares.

### 2.5 Infant brains

To quantify thickness variations throughout the first two years of life, we acquire magnetic resonance images of *n* = 73 healthy infants, shortly after birth and one and two years later, using a Siemens head-only 3T scanner (Allegra, Siemens Medical System, PA) with a circular polarized head coil. All scans are performed in accordance with the guidelines and regulations of the University of North Carolina’s Institutional Review Board, which approved this study. Pregnant mothers are recruited during the second trimester of pregnancy and informed consent was obtained from both parents. We pre-process all images using our established automatic, infant-specific computational pipeline [47]. Segmenting neonatal brain scans is a challenging problem because of their poor tissue contrast and their large within-tissue intensity variability.

**Fig. 3.**
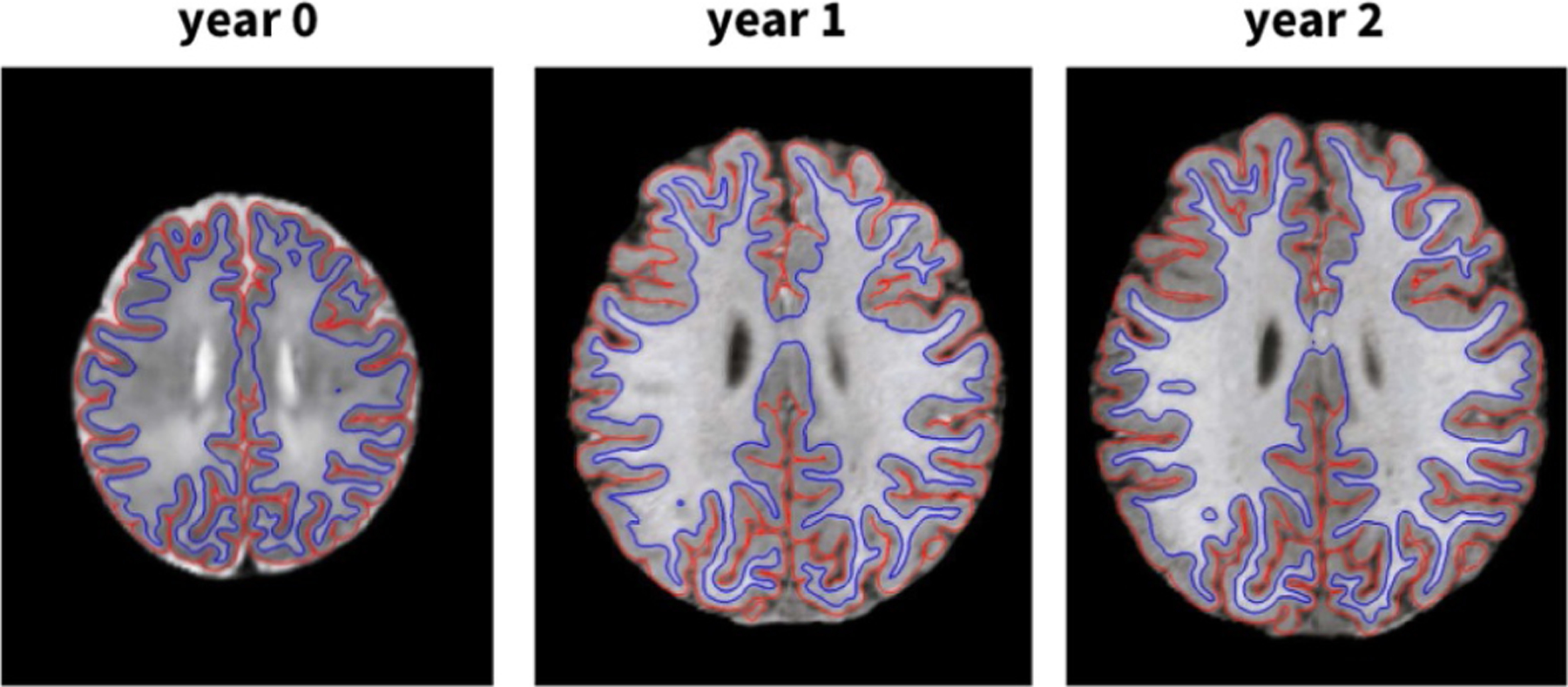
Infant brains. Reconstructed longitudinal outer (red) and inner (blue) cortical surfaces of a representative infant brain at years 0, 1, and 2 generated by the infant-specific computational pipeline for surface-based analysis.

To address this issue, we employ a novel image analysis approach that utilizes subject-specific atlases, which allow for longitudinal registration and segmentation. For each infant, we perform a nonlinear image registration of the neonatal and one-year old scans onto the associated two-year old scan, which typically has much better image contrast and clear cortical folding structures [48]. This strategy significantly mitigates ambiguity, increases segmentation accuracy and longitudinal consistency, and greatly facilitates cortical surface reconstruction in early infancy [49]. Figure 3 illustrates how we reconstructed the cortical surface using our infant-specific computational pipeline for surface-based analysis, which we have extensively verified on more than 500 infant brain images [50]. We identify gyral and sulcal regions using a hidden Markov random field model and an expectation maximization algorithm on the maximum principal curvatures of the cortical surface. To parcellate each cortex into four lobes, we manually delineate the temporal, frontal, parietal, and occipital lobes on the surface atlases based on a parcellation protocol [51] and propagate labels from the surface atlases onto each individual cortical surface.

### 2.6 Adult brains

To quantify thickness variations across the human brain, we use magnetic resonance images of *n* = 9 healthy adult human brains at the Stanford University Center for Cognitive and Neurobiological Imaging (CNI) using a 3Tesla scanner (GE MR750, Milwaukee, WI) with a 32-channel radiofrequency receive head coil (Nova Medical, Inc., Wilmington, MA) [52]. All scans are performed in accordance with the guidelines and regulations of Stanford University’s Institutional Review Board, Human Subjects Division, which approved all the experimental protocol and procedures. Written informed consent is obtained from every participant in the study. We perform volumetric image segmentation and cortical reconstruction using FreeSurfer (Harvard University, Cambridge, MA) [53]. To parcellate the cortical surface into gyral and sulcal regions, we adopt the Destrieux atlas [54], which is implemented in FreeSurfer as an automatic, surface-based parcellation and labeling method based on widely-accepted anatomical conventions [33]. The limits of each region are determined through a probabilistic labeling process, taking into account the local mean curvature and convexity, and labels of neighboring vertices. This resulted in the separation of gyri and their neighboring sulci. In some instances, the parcellations are small or difficult to localize; these regions are excluded from our analysis since they cannot be classified as exclusively gyral or sulcal. For all remaining anatomic regions, we report gyral and sulcal thicknesses as area-weighted values to account for regional size variations and for the fact that large brains tend to be significantly more folded than small brains [55]. To compare our own brain scans to a public data base, we analyze magnetic resonance images of *n* = 564 healthy adult human brains [56]. We excluded nine files from the original data set because of incomplete information. Similar to analyzing our own scans, we perform volumetric image segmentation and cortical reconstruction using FreeSurfer [53] and parcellate the cortical surface into 58 gyral and 62 sulcal regions [33]. We determine the cortical thickness in each of these 67 680 regions and report the gyral and sulcal thicknesses both individually for each lobe and collectively in a histogram. Figure 1 shows the normalized averaged gyral and sulcal human brain thicknesses in all four lobes as red and blue triangles.

## 3 Results

### 3.1 Gyral thickness is significantly larger than sulcal thickness

**Fig. 4.**
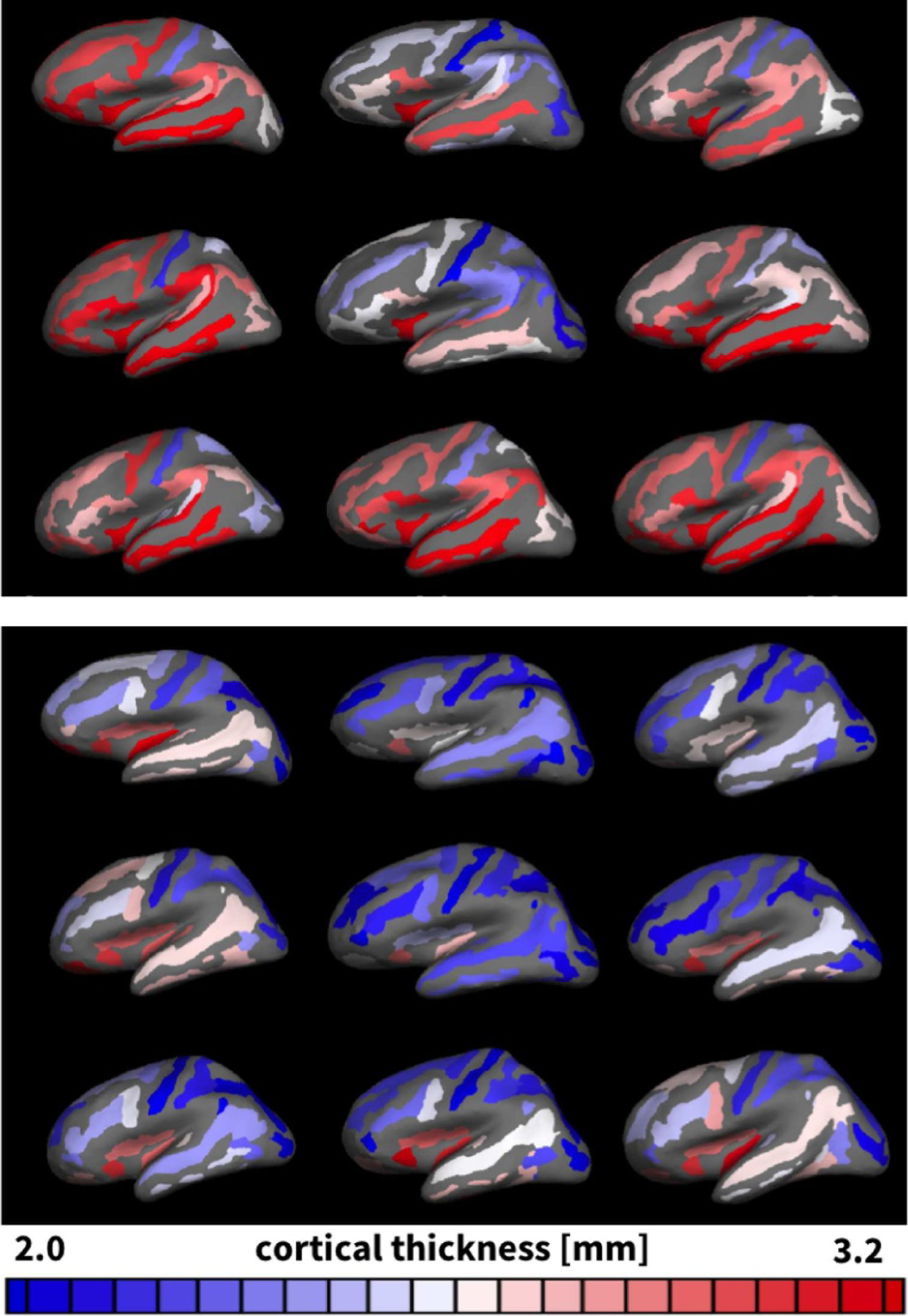
Thickness variations in adult human brains. Gyral (top) and sulcal (bottom) regions of *n* = 9 adult human brains reconstructed, parcellated, and inflated to display the complete pial surface. Regions are color-coded according to the average gyral and sulcal thicknesses in each region; all remaining regions are shown in gray.

**Fig. 5.**
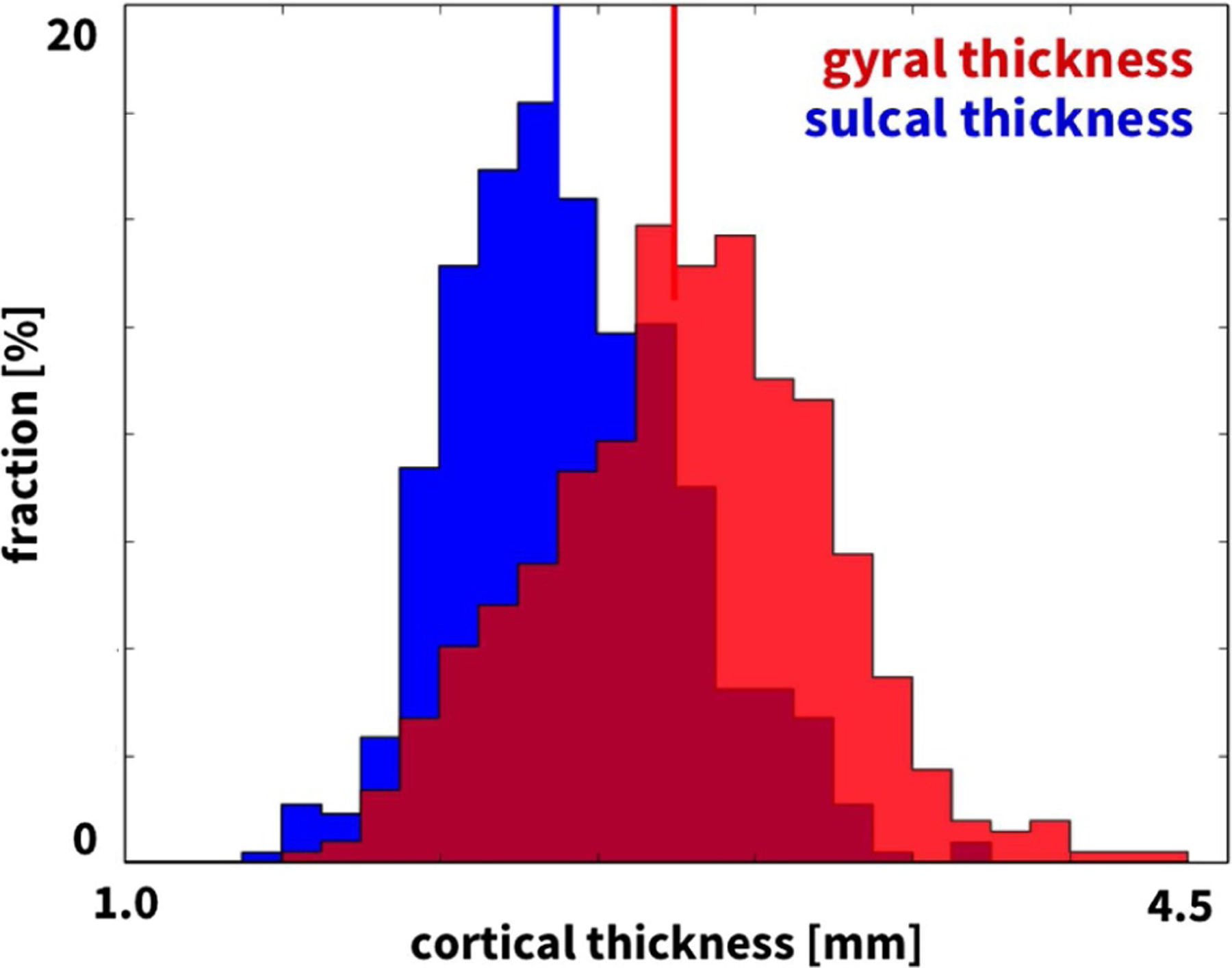
Thickness variations in 9 adult human brains. Histogram of gyral (red) and sulcal (blue) thicknesses of 58 gyral regions and 62 sulcal regions for our *n* = 9 adult human brains, see Figure 4. Vertical lines indicate the average gyral and sulcal thicknesses of 2.74 mm and 2.37 mm.

**Fig. 6.**
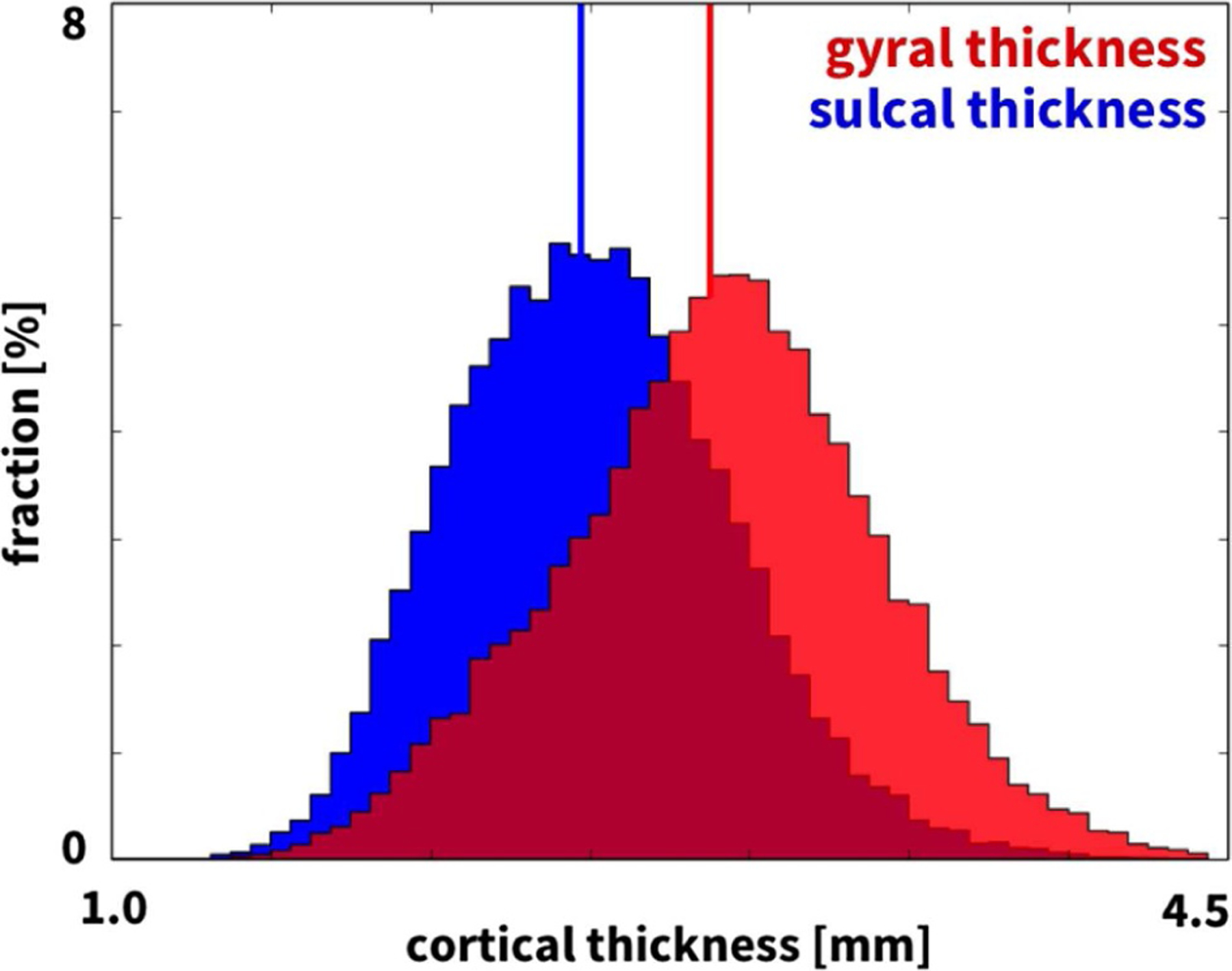
Thickness variations in 564 adult human brains. Histogram of gyral (red) and sulcal (blue) thicknesses of 58 gyral regions and 62 sulcal regions for *n* = 564 healthy adult human brains. Vertical lines indicate the average gyral and sulcal thicknesses of 2.87 mm and 2.47 mm. Figure adopted from [20].

Figure 4 illustrates the reconstructed, parcellated, and inflated gyral (top) and sulcal regions (bottom) of *n* = 9 healthy adult human brains. The color-code highlights the regional variation in cortical thickness ranging from 2.0 mm shown in blue to 3.2 mm shown in red. Figure 5 summarizes the thicknesses of 29 gyral and 31 sulcal regions of the left and right hemispheres for our *n* = 9 brains in a cortical thickness histogram. The red and blue vertical lines indicate the average area-weighted gyral and sulcal thicknesses. The gyral thickness of 2.74 mm was larger than the sulcal thickness of 2.37 mm with an overall gyral-to-sulcal thickness ratio of 1.157. For comparison, Figure 6 illustrates the gyral and sulcal thicknesses for the *n* = 564 healthy adult human brains from the public database [56]. In agreement with our *n* = 9 brains in Figure 5, for the *n* = 564 brains in Figure 6, the gyral thickness of 2.87 mm was larger than the sulcal thickness of 2.47 mm with an overall gyral-to-sulcal thickness ratio of 1.162.

### 3.2 Gyral and sulcal thicknesses increase from back to front

**Fig. 7.**
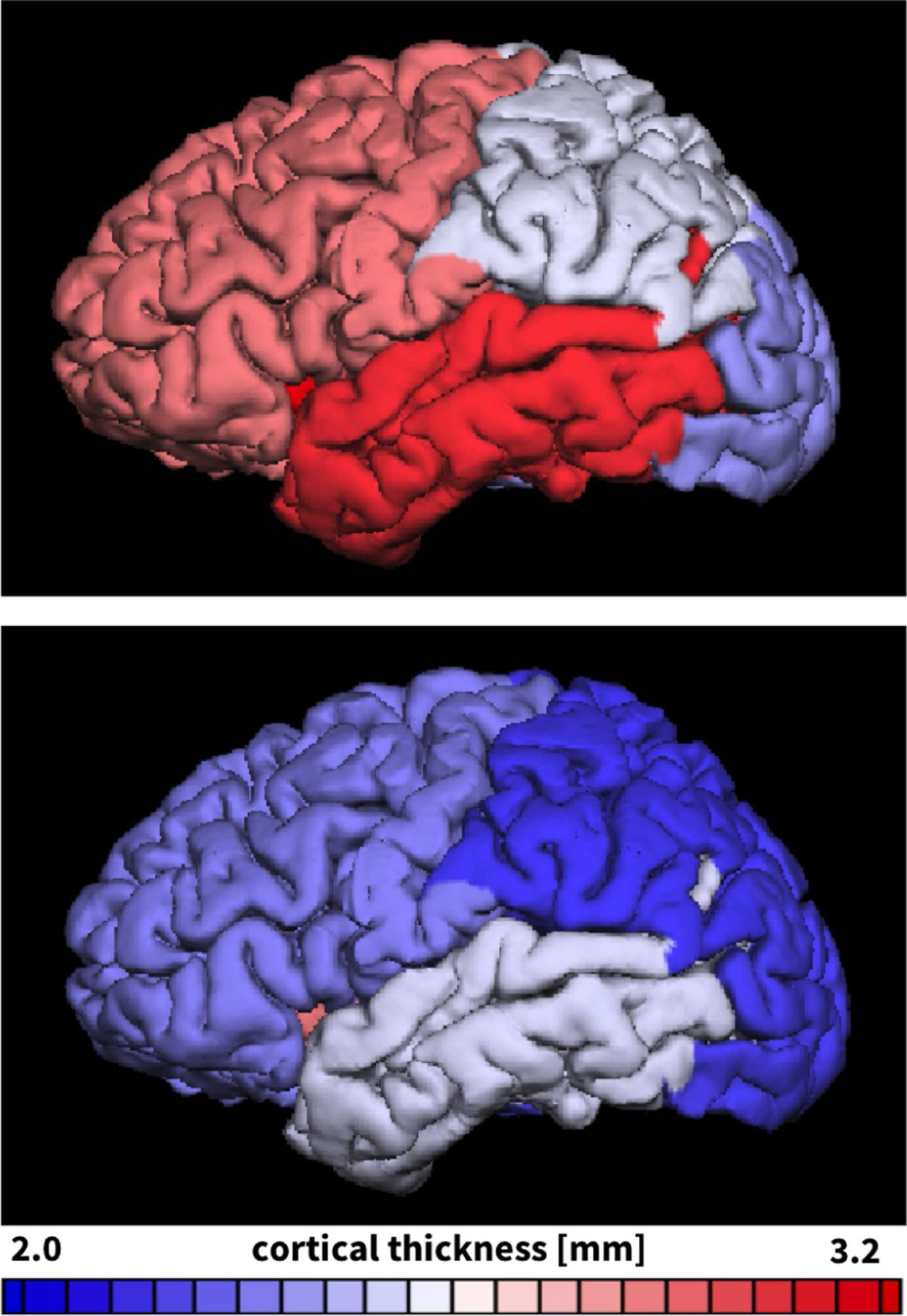
Thickness variations in adult human brains. Gyral (top) and sulcal (bottom) thicknesses of the temporal, frontal, parietal, and occipital lobes averaged over *n* = 9 adult human brains and collectively displayed on one of the brain surfaces of Figure 4. Figure modified from [20].

Figure 7 summarizes the gyral and sulcal cortical thicknesses of the temporal, frontal, parietal, and occipital lobes averaged over our *n* = 9 brains, collectively displayed on one of the brain surfaces of Figure 4. The temporal cortex was thickest with gyral values of 3.06±0.21 mm and sulcal values of 2.54±0.15 mm; the occipital cortex was thinnest with gyral values of 2.40±0.12 mm and sulcal values of 2.21±0.12 mm. The thickness ratio between gyri and sulci was larger in the frontal and temporal lobes with 1.218 and 1.206 than in the parietal and occipital lobes with 1.167 and 1.082. The thickest region was region 18 of the Destrieux atlas [54], the short insular gyrus, with 3.75±0.71 mm and the thinnest region was region 57, the middle occipital sulcus and lunatus sulcus, with 1.89±0.42 mm.

**Fig. 8.**
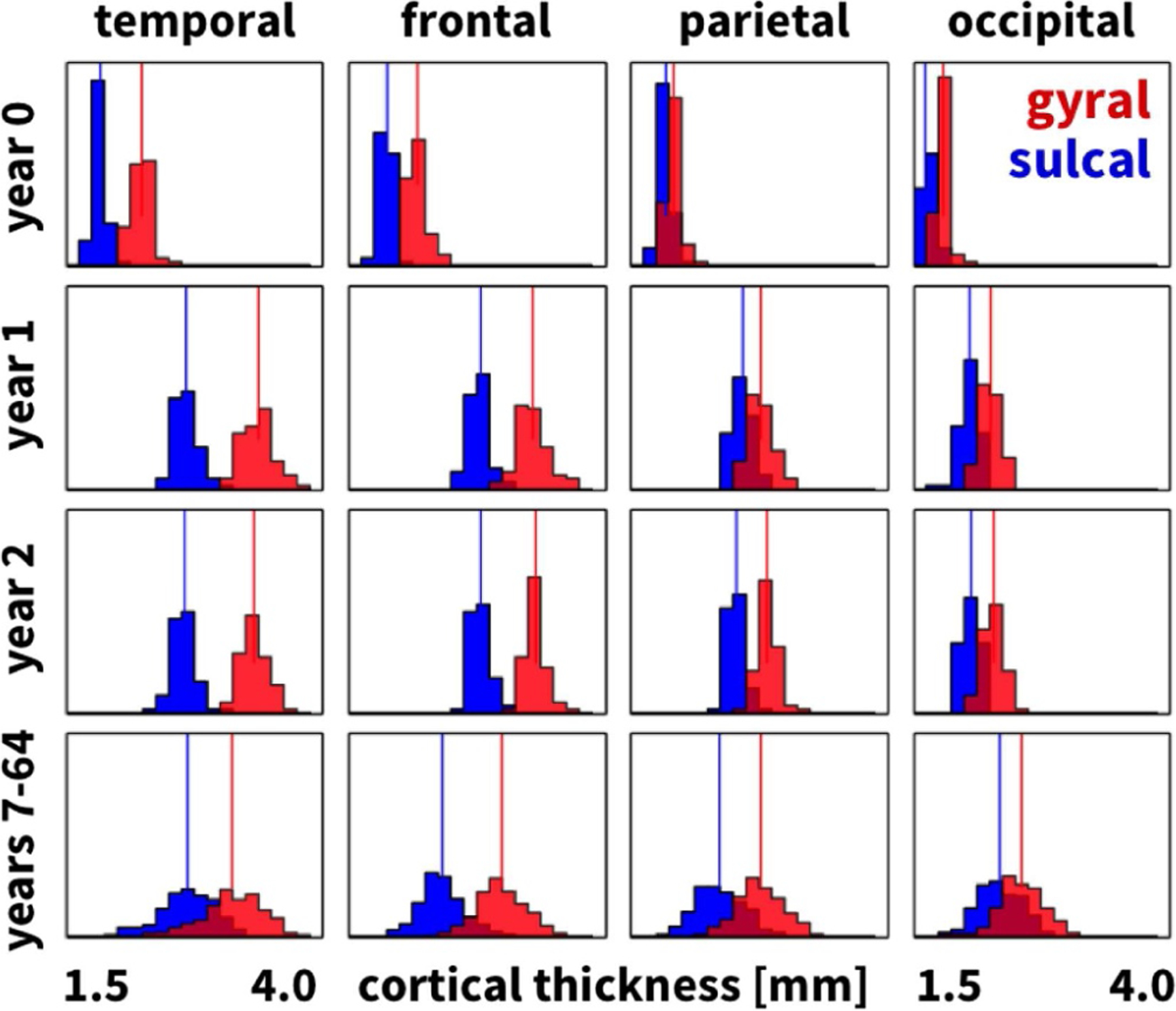
Thickness variations in infant and adult human brains. Histograms of gyral (red) and sulcal (blue) thicknesses in temporal, frontal, parietal, and occipital lobes for *n* = 73 infant brains, each scanned at years 0, 1, and 2, and, for comparison, for *n* = 564 healthy brains between years 7 and 64. Vertical lines indicate the average gyral and sulcal thicknesses.

Figure 8 and Table 1 summarize the gyral and sulcal cortical thicknesses of the temporal, frontal, parietal, and occipital lobes for *n* = 73 infant brains, each scanned in a longitudinal study at years 0, 1, and 2 [49], and for comparison, for *n* = 564 healthy brains between ages 7 and 64 years [56]. In agreement with our *n* = 9 brains in Figure 7, the temporal and frontal cortices of the *n* = 73 infant brains and the *n* = 564 brains in Figure 8 are markedly thicker than the parietal and occipital cortices. Strikingly, the gyral and sulcal cortices of all four lobes thicken notably between years 0 and 1, but do not change significantly between years 1 and 2. Mean cortical thicknesses only display marginal differences between years 1 and 2 compared to the large data set of years 7–64; standard deviations, however, more than double for the widely spread age group from 7 to 64 years. All gyral thicknesses are significantly larger than the corresponding sulcal thicknesses $\left(p\ll 10^{-10}\right)$.

### 3.3 Gyral-to-sulcal thickness ratio increases with fold size

**Fig. 9.**
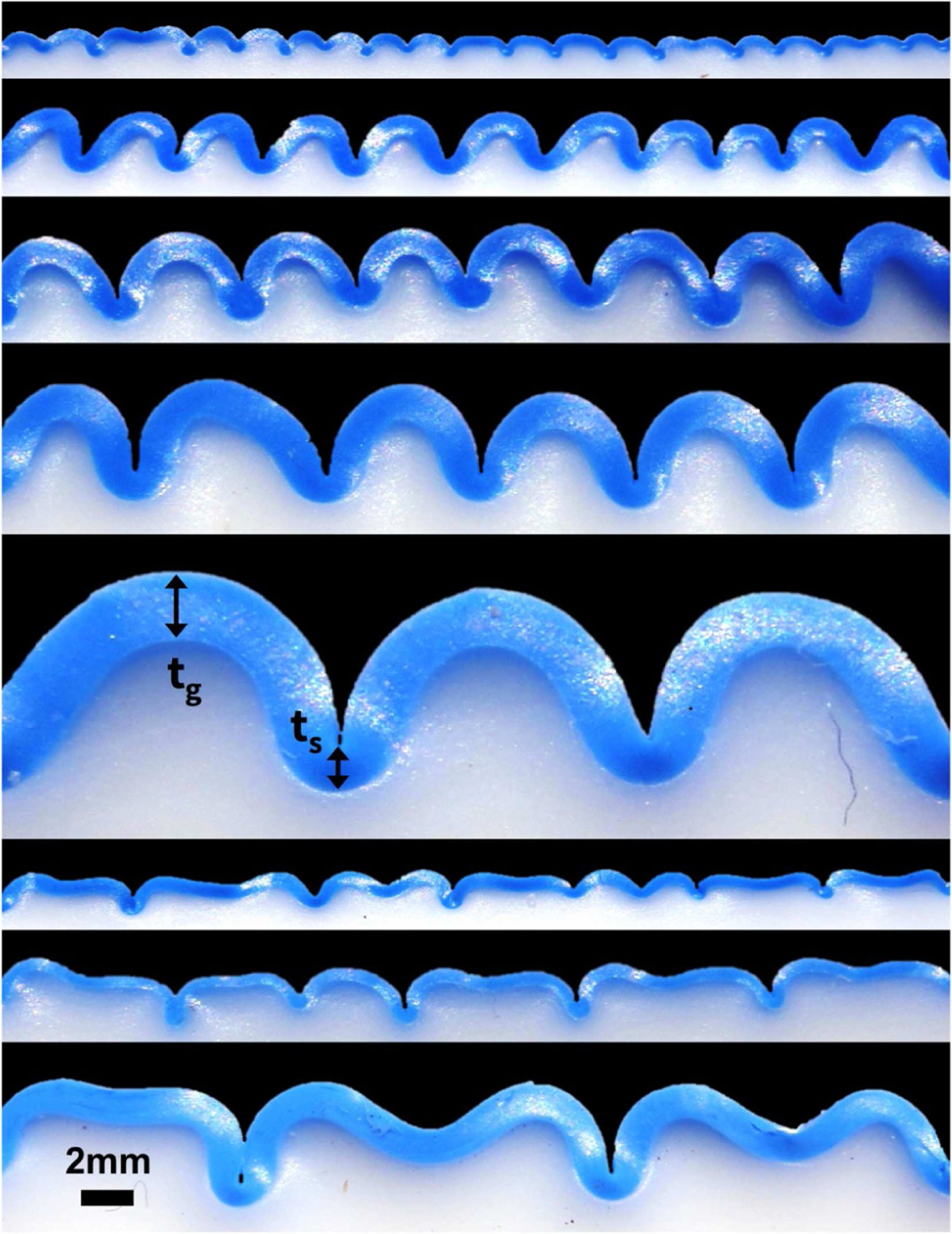
Thickness variations in polymer model. Folding patterns of regular folding for varying initial layer thicknesses of 0.30 mm, 0.60 mm, 0.80 mm, 1.00 mm, and 2.00 mm and period doubling for layer thicknesses of 0.45 mm, 0.60 mm, and 1.00 mm, from top to bottom, at constant prestretch of λ = 2.0. Figure (top) adopted from [20].

Figure 9 and Table 2 illustrate the emerging gyral and sulcal thicknesses in our bilayered polymeric gel model upon releasing a prestretch of λ = *l/L* = 2.0. At this prestretch, the stiff thin layer dyed in blue had a stiffness of 324 kPa and the soft thick substrate dyed in white had a stiffness of 69 kPa. With these values, the layer and substrate of our polymer model are 75 and 35 times stiffer than the adult human brain with a Young’s modulus of 4.29 kPa in the gray matter layer and 1.98 kPa in the white matter substrate [45]. Our model stiffness ratio of 4.7 between layer and substrate is about twice as large as the stiffness ratio of three observed in the adult human brain.

Varying the initial layer thicknesses between 0.30 mm, 0.60 mm, 0.80 mm, 1.00 mm, and 2.00 mm confirms the generally accepted notion that the wavelength scales linearly with the layer thickness [57]. In addition, our polymer experiments suggest that the thickness ratio between gyri and sulci increases with increasing initial thickness and fold size from 1.274 for an initial layer thickness of 0.30 mm, via 1.490 for a layer thickness of 0.60 mm, 1.543 for a layer thickness of 0.80 mm, and 1.589 for a layer thickness of 1.00 mm, to 1.622 for a layer thickness of 2.00 mm. For the mode of period doubling illustrated in the last three rows of Figure 9 and Table 2, we observed similar trends with thickness ratios between gyri and sulci of 1.277 for a layer thickness of 0.45 mm, 1.295 for a layer thickness of 0.60 mm, and 1.330 for a layer thickness of 1.00 mm.

**Fig. 10.**
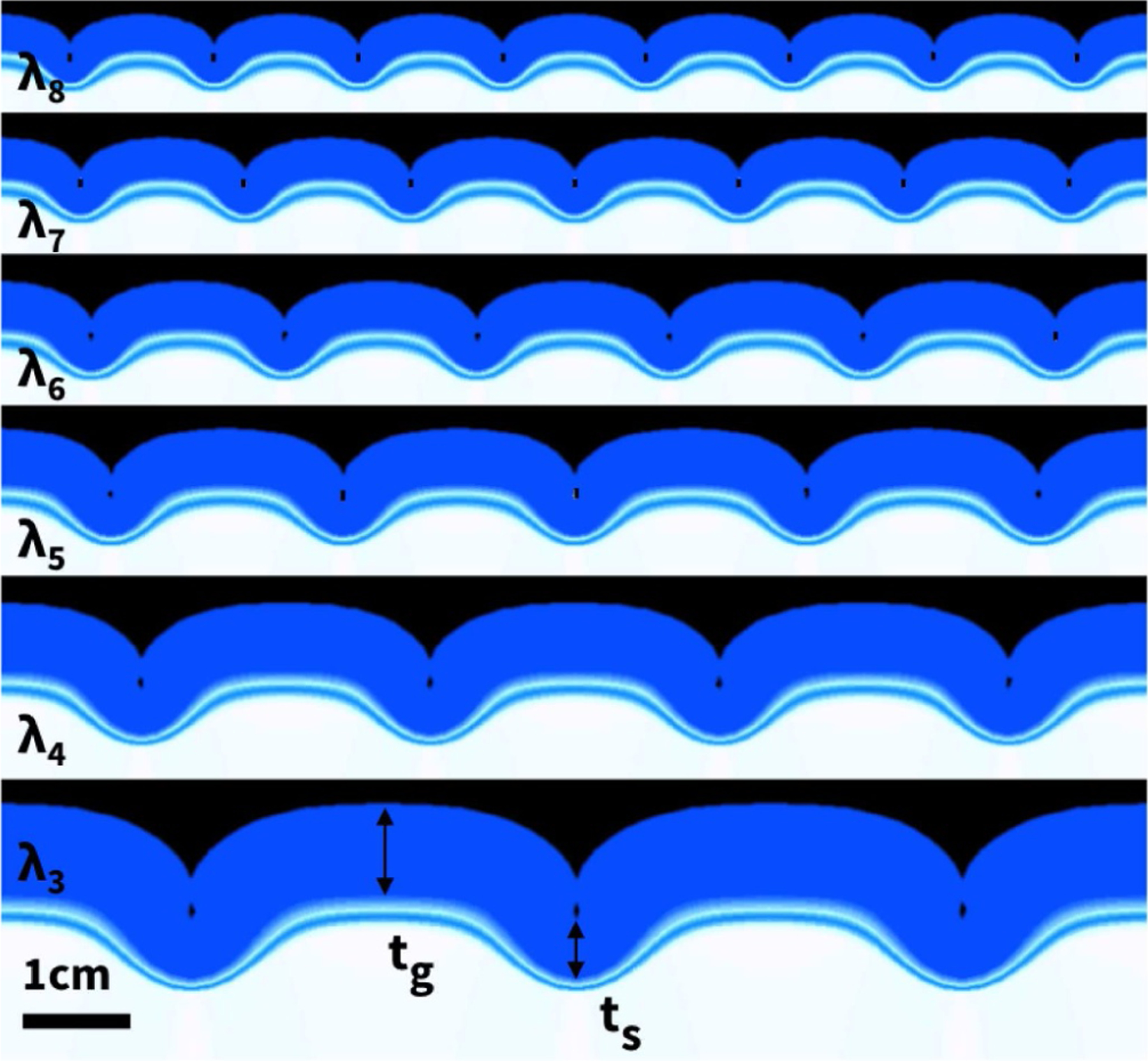
Thickness variations in computational model. Folding patterns for varying cortical thicknesses of 1.25 mm, 1.43 mm, 1.67 mm, 2.00 mm, 2.50 mm and 3.33 mm, from top to bottom, at first point of self-contact, at an average growth of *ϑ* = 1.843 ± 0.013. Figure adopted from [20].

Figure 10 illustrates our computational simulation of the bilayered model system with varying initial layer thicknesses of 1.25 mm, 1.43 mm, 1.67 mm, 2.00 mm, 2.50 mm and 3.33 mm. The simulations predict a similar trend as the experiment in Figure 9 and confirm that the wavelength scales linearly with the layer thickness, here increasing from 12.0 mm, 13.7 mm, 16.0 mm, 19.2 mm, and 24.0 mm, to 32.0 mm at a constant thickness-to-wavelength ratio of 0.104. The six snapshots illustrate the point of first self-contact, at an average growth factor or *ϑ* = 1.843 ± 0.013.

### 3.4 Gyral-to-sulcal thickness ratio increases with growth

**Fig. 11.**
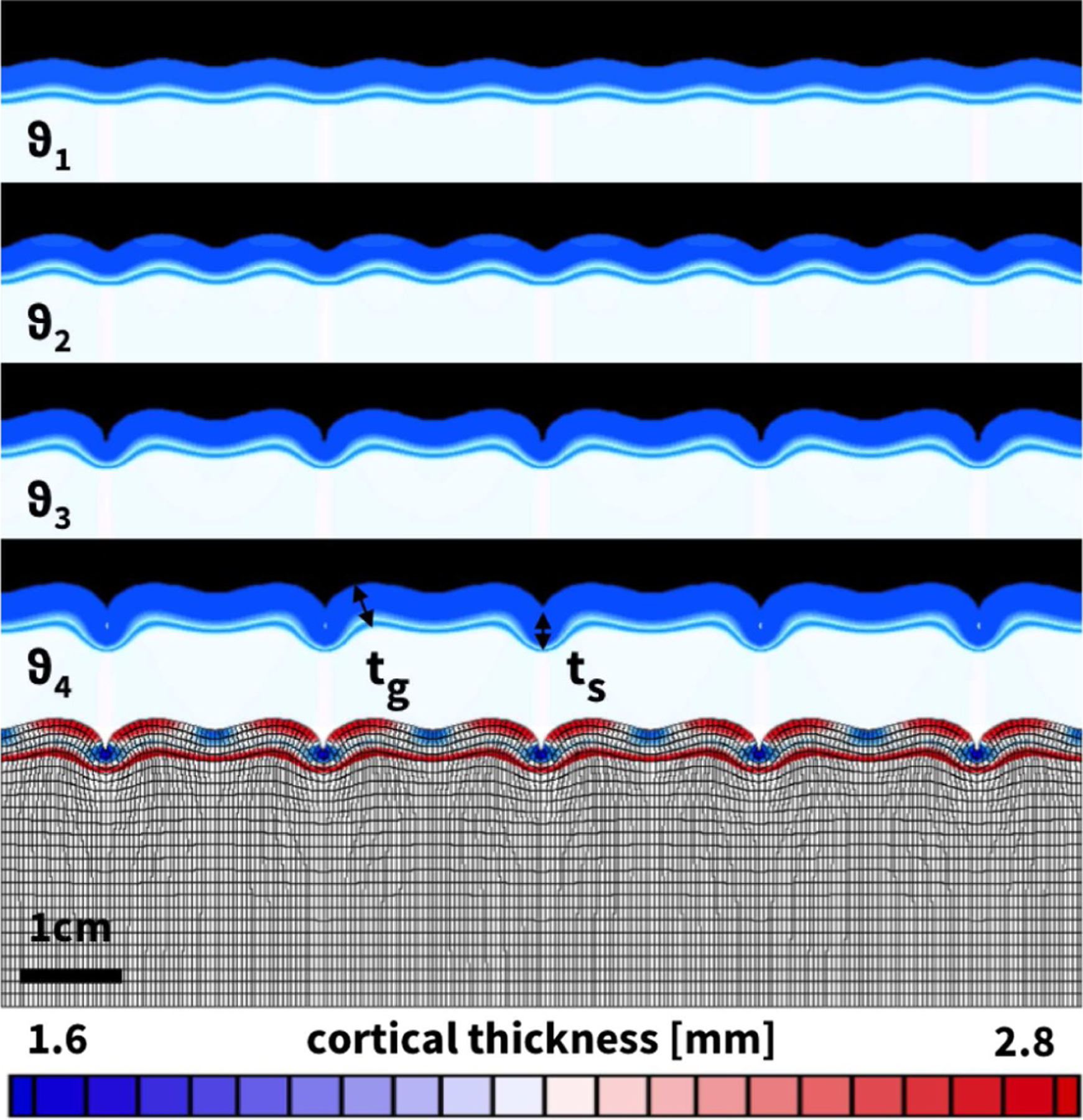
Thickness variations in computational model. Emerging folding patterns: Moderate growth beyond the first instability point creates a symmetric, sinusoidal folding patterns at *ϑ*_1_ = 1.692. Further growth triggers symmetry breaking into a non-symmetric folding pattern with sharper sulci and smoother gyri at *ϑ*_2_ = 1.735. Continuing growth beyond a second instability point initiates period-doubling with alternating increasing and decreasing sulci at *ϑ*_3_ = 1.776. As growth continues, contact zones emerge along two neighboring edges of increasing sulci, while decreasing sulci have almost entirely flattened out at *ϑ*_4_ = 1.883.

Figure 11 and Table 3 summarize the evolving gyral and sulcal thicknesses in our computational model system upon gradual layer growth. In contrast to the prestretch experiment, the growth simulation allows us to explore both the final folding pattern and the progressive evolution of pattern formation. Figure 11 highlights four representative time points associated with symmetric folding with sinusoidal modes, non-symmetric folding with sharper sulci and smoother gyri, period-doubling with alternating increasing and decreasing sulci, and contact of neighboring edges of increasing sulci, while decreasing sulci have almost entirely flattened out [58]. The gyral thickness increases gradually from 2.52 mm to 2.84 mm, while the sulcal thickness decreases from 2.41 mm to 1.58 mm. The bottom row highlights the predicted cortical thickness varying from 2.84 mm in the gyral regions shown in red to 1.58 mm in the sulcal regions shown in blue.

**Fig. 12.**
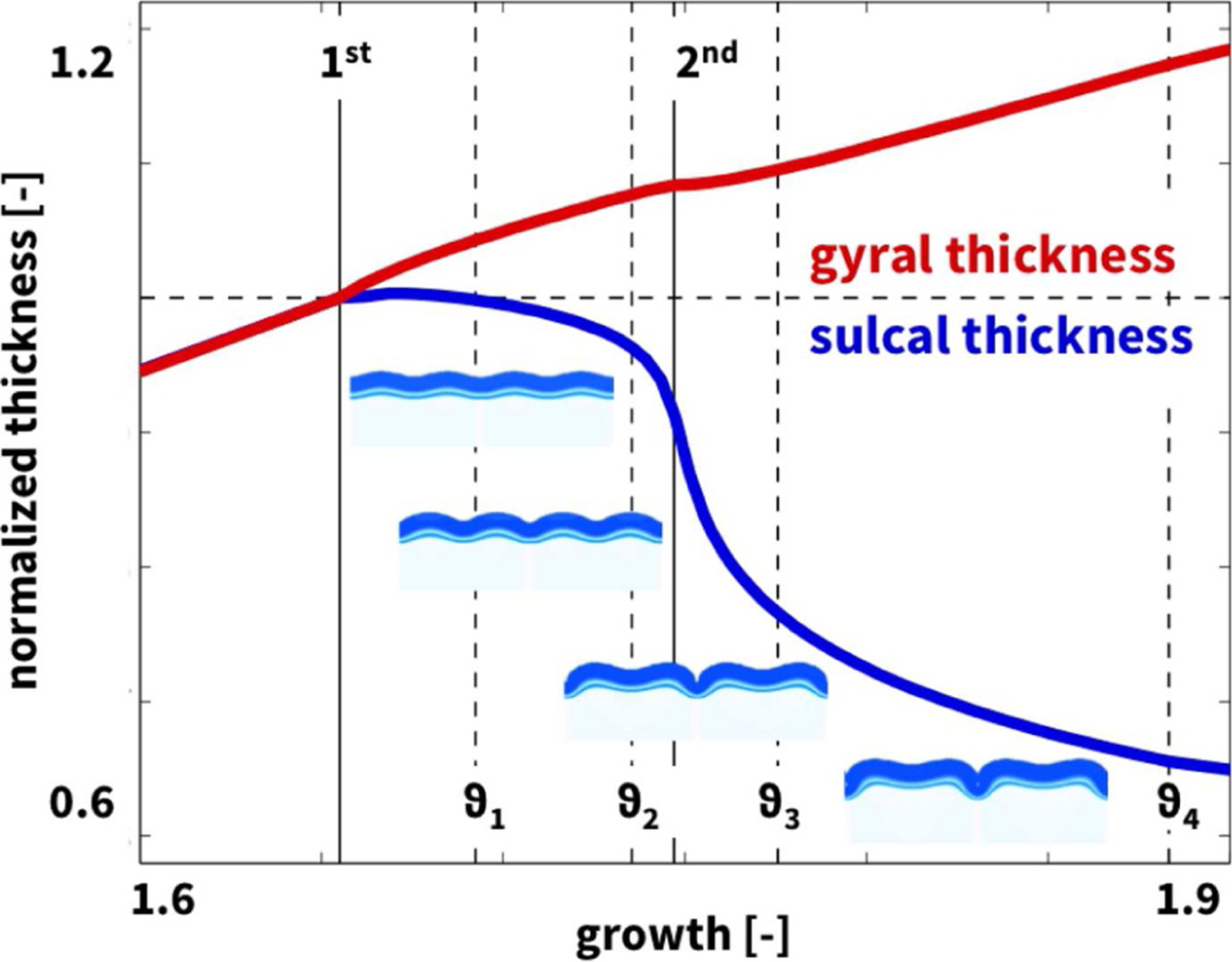
Thickness variations in computational model. Emerging normalized gyral (red) and sulcal (blue) thicknesses after first bifurcation at a layer growth of *ϑ* = 1.655 and second bifurcation at *ϑ* = 1.747 (solid lines); *ϑ*_1_ = 1.692, *ϑ*_2_ = 1.735, *ϑ*_3_ = 1.776, and *ϑ*_4_ = 1.883 (dashed lines) correspond to the time points in Figure 11.

Figure 12 illustrates the evolving gyral and sulcal thicknesses as the layer continues to grow. Both thicknesses remain identical until the first bifurcation point at *ϑ* = 1.655. In the post-bifurcation regime, the gyral thickness increases while the sulcal thickness decresaes. After the second bifurcation point at *ϑ* = 1.747, beyond the onset of period doubling, the sulcal thickness drops drastically until *ϑ* = 1.883 when two neighboring folds begin to form contact. Our computational simulations suggest that the thickness ratio between gyri and sulci increases as growth progresses from 1.000 at the first bifurcation point to 1.430 at the second bifurcation point to 1.790 at the point of self-contact.

## 4 Discussion

### 4.1 Cortical thickness varies markedly across the human brain

In his famous 1908 monograph *Über Rindenmessungen*, the German neurologist Korbinian Brodmann was the first to acknowledge that the cortical thickness displays significant variations across the brain [59]. We now know that measurements of the cortical thickness play an important role in normal development and can serve as biomarkers in a wide variety of neurodegenerative and psychiatric disorders: Alterations in cortical thickness are common in normal aging, but also closely associated with Alzheimer’s disease, dementia, Huntington’s disease, amyotropic lateral sclerosis, and schizophrenia. A recent study suggests that the cortical thickness and surface area are directly correlated to the degree of folding [9]. Our *n* = 9 analyzed brains support the recently proposed universal scaling law [10], $k\;A_{\text{e}}^{5/4}/A_{\text{t}}=t_{\text{c}}^{1/2}$ which relates the exposed surface area *A*_e_, the total surface area *A*_t_, and the cortical thickness *t*_c_ through a dimensionless scaling parameter *k* that varies with age and disease. Remarkably, our brains in Figure 4 have an exposed surface area of *A*_e_ = 594±30 cm^2^, a total surface area of *A*_t_ = 1791±156 cm^2^, and a cortical thickness of *t*_c_ = 2.58±0.14 mm, resulting in a scaling parameter of *k* = 0.310±0.007 with a standard deviation as low as 2%. The theory postulates that *k* is related to the cortical tension [10], a physics-based metric that changes with alterations in cerebrospinal fluid pressure [9] and white matter stiffness [45]. A point of criticism of this theory that compared cortical folding to crumpling a piece of paper [9] is that its thickness remains constant and does not change to reflect growth [24]. Here we studied the dynamics of cortical folding, analytically and computationally in a continuously growing layer on an ultrasoft elastic substrate [60] and experimentally in a prestretched polymeric bilayer [61]. In all cases, in Figure 1 and Figures 9–12, the homogeneous bilayered system undergoes a unique symmetry breaking into a spatially heterogeneous system with discrete gyri and sulci. These observations agree with the trends observed in a computational model, a swelling gel model, and histological slices of mammalian brains [11]. While the above scaling law accounts for the cerebrospinal fluid phenomenologically through the scaling parameter *k*, in our model, we could rigorously include the cerebrospinal fluid by adding an external pressure on the growing upper layer. This would compress the bilayered system and create a state of tension in the lateral direction that would delay the onset of the instability and create flatter morphologies, while the emerging wavelengths would remain unaffected [11].

A more important parameter than the external pressure is the stiffness ratio between gray and white matter [62]. There seems to be a general agreement that the stiffness ratio in the adult human brain is on the order of one [11,63], with reported values of 2.17 for triaxial testing ex vivo [64] and 1.15 for magnetic resonance elastography in vivo [65]. Those values are on the order of 1.54 reported for rat tissue slices [66], and slightly larger than those of 1.04 for porcine tissue blocks [67], 0.73 for bovine tissue slices [68], and 0.67 for porcine tissue slices [69] ex vivo. Recent studies have shown though that the white matter stiffness increases linearly with the degree of myelination and that it is three times smaller in the pre-natal brain than in the post-natal brain [70]. Without further studies, it remains unclear whether gray or white matter is stiffer in the living fetal human brain at the onset of cortical folding [71]. Our observed symmetry breaking, however, is universally valid for stiffness ratios above the creasing threshold [63]: For model systems of two-dimensional prestretch or growth, the critical stiffness ratio for zero-wavelength Biot instabilities is 0.35 [72], between 0.35 and 0.86 we expect creasing, and above 0.86 we expect folding [19]. This explains why, in regions of the brain with gray-to-white matter stiffness ratios above the creasing threshold, the emerging patterns are similar to our analytical and computational models with stiffness ratios of 3 and to our experimental model with 21. Interestingly, a recent study suggests that folding is still possible for smaller stiffness ratios provided we model the brain as a trilayer system [73]. Strikingly, in all four model systems, analytical, computational, polymer, and organoid, as growth progresses, the *gyri universally thicken* while the *sulci universally thin*, even in the complete absence of regional information.

### 4.2 Genetic, geometrical, and physical factors modulate shape

Interestingly, a recent study attributed regional differences in cortical development exclusively to genetic organization patterns [74]. While genetic effects obviously play a role in brain development, both in the human brain and likely in the organoid models in Figure 2, our study demonstrates that geometric and physical factors are sufficient to produce cortical patterning and modulate cortical thickness [20]. This suggests that genetic, geometric, and physical factors during brain development are closely coupled. The observations included here and in [20] agree well with the *mechanical feedback hypothesis*, which suggests that mechanical cues are part of the body’s complex signaling process by which growth is directed [75]. This question is closely related to the role of mechanical instabilities in fingered growth phenomena [76] or tumor growth [77]. For the brain, understanding regional and temporal variations in cortical thickness can provide insight into the evolution and causative factors of a disease, and help us assess the efficacy of a wide variety of treatments [78]. For example, abnormal circuitry in epilepsy patients is often restricted to small cortical abnormalities, e.g., a local thickening of the sulcal fundus [32] and can be surgically cured by removing the thickened region [79]. A better understanding of cortical thickness ratios and of the limitations associated with magnetic resonance imaging will allow us to detect cortical abnormalities more precisely and perform in vivo diagnostics of epilepsy.

### 4.3 Gyral thickness is significantly larger than sulcal thickness

Although we have known for almost a century that the cortical thickness is typically larger in gyri than in sulci [80,81], it remains unclear whether these thickness variations are the cause or consequence of cortical folding. Our *n* = 9 gyral and sulcal thicknesses of 2.74 mm and 2.37 mm in Figure 5, the gyral and sulcal thicknesses of the *n* = 564 comparison brains of 2.87 mm and 2.47 mm in Figure 6, and the gyral and sulcal thicknesses of our *n* = 73 infant brains of 2.92 mm and 2.52 mm at year 1 and of 2.94 mm and 2.50 mm at year 2 in Figure 8 agree well with the reported gyral and sulcal thickness of 2.7 mm and 2.2 mm averaged over 30 subjects [33]. Within a month after birth, however, our *n* = 73 infant brains displayed significantly thinner gyral and sulcal cortices of 2.03 mm and 1.78 mm. In comparison to a recent study that reported gyral-to-sulcal thickness ratios of 1.6, 1.6, and 2.0 measured in two-dimensional histological slices of porcupine, cat, and human brains [11], our three-dimensional magnetic-resonance-based thickness ratios from 1.044 to 1.263 in Table 1 are slightly lower. This discrepancy could result from the fact that magnetic resonance imaging is only a proxy of the real cortical thickness as measured in histological slices; yet, at the advantages of being non-invasive, automatable, and readily applicable to neonatal brains [50]. Unfortunately, however, T1- and T2-weighted magnetic resonance images display an extremely poor tissue contrast during development, which makes cortical thickness measurements based on the tissue contrast of a single time point less reliable [49]. Our current analysis uses a set of infant-specific computational techniques that allows us to characterize the cortical thicknesses by capitalizing on longitudinal T1- and T2-weighted magnetic resonance images during infancy [47]. Yet, with current imaging techniques, characterizing cortical thicknesses at the onset of folding, e.g., in very preterm neonates, remains challenging if not impossible. Recent studies found a linear trend between the gyral and sulcal cortical thickness, both in a computational model and in a real human brain [82]. Our study confirms these general trends [83]: We not only provide a *static picture* of the thickness ratio in adult human brains in Figures 4 and 7, at the final stage of the polymer experiment in Figure 9 and Table 2, and at the end of the computational simulation in Figure 10; we also show that both the thickness and the thickness ratio between gyri and sulci *evolve dynamically* and increase continuously with increasing growth, through a longitudinal analysis of magnetic resonance images in Figure 8 and Table 1, computationally in Figures 11 and 12 and Table 3, and analytically in Figure 1. This could explain why the gyral-to-sulcal thickness ratio is larger in the frontal lobe with a lower cortical thickness than in the temporal lobe. Association areas continue to develop and grow several months after birth, longer than other brain regions [84]. This agrees well with our cortical thickness growth in Table 1: The temporal and frontal cortex thickened on average by 50.8% and 51.6%, the parietal cortex by 44.2%, and the occipital cortex by 25.2% within the first year after birth. Our findings could indicate that brain regions associated with higher cognitive functions have a higher gyral-to-sulcal thickness ratio; in other words, the thickness ratio could provide an indication of how advanced certain brain regions are. Further evidence is needed to support this claim, e.g., by mapping sets of primary and higher functions onto distinct brain regions and correlating them to the corresponding thickness values. Our dynamic growth simulations reveal that beyond the first bifurcation point, the gyral thickness keeps growing almost linearly with increasing growth, whereas the sulcal thickness drops drastically and then converges to less than 65% of its initial value, see Figure 12. This dramatic change in the mechanical environment – both in cortical thickness and in cortical tension – could induce mechanical feedback at the cellular level [26] and explain the differential expression of genes that regulate later development [24].

### 4.4 Anterior thickness is larger than posterior thickness

More than a century ago, studies of the human cortex have revealed significant regional variations in thickness ranging from 1.5 to 4.5 mm [36], values which agree well with our histogram in Figure 5. Our cortical thickness histogram for 120 brain regions of all nine brains displays similar characteristics as a cortical thickness histogram for a single triangulated brain surface [33], but, in addition, also highlights a significant difference between the thicknesses in the 58 gyral and 62 sulcal regions. In addition to gyral-sulcal thickness variations, our brains also display an anterior-posterior thickness gradient, with thickest regions in the frontal and temporal gyri and thinnest regions in the parietal and occipital sulci, see Figure 7. Although magnetic-resonance-based cortical thickness measurements are only a proxy of the real cortical thickness as measured in histological slices, our thickness gradients agree remarkably well with the early histological measurements by Korbinian Brodmann, who reported thickest regions in the temporal and frontal lobes and thinnest in the parietal and occipital lobes [59]. Recent comparative analyses of cortical folding have argued that the rostro-caudal axis of the central nervous system is the main direction along which mammalian neuroanatomical diversity is organized [85]: Early differentiated posterior regions are more folded than late differentiated anterior regions [55]. Our analytical model, our computational model in Figure 10, and our experimental model in Figure 9 support this observation that the thinner posterior regions are more folded than the thicker anterior regions. This agrees well with the first fully automated characterization of the cortical thickness, which identified Brodmann’s area 4 on the anterior bank with more than 4 mm as the thickest region and Brodmann’s area 3 on the posterior bank of the central sulcus with less than 2 mm as the thinnest region [33]. For our nine brains, the thickest region was region 18 of the Destrieux atlas [54], the short insular gyrus, with 3.75±0.71 mm and the thinnest region was region 57, the middle occipital sulcus and lunatus sulcus, with 1.89±0.42 mm. Interestingly, the thickest regions of our brain, the lateral temporal cortex, the temporal pole, and the precentral gyrus with average thicknesses of 3.5–4.5 mm are also the fastest expanding regions during development, whereas the thinner regions like the visual cortex with a thickness of 2.0 mm are the slowest expanding regions [86].

### 4.5 Thickness variations emerge naturally in bilayered systems

When an initially homogeneous layer folds, symmetry breaking causes gyri to thicken and sulci to thin [20]. Thickness asymmetries originate at the first instability point of sinusoidal folding and increase dynamically towards the second instability point of period doubling [58]: the larger the amount of growth, the larger the gyral-to-sulcal thickness ratio, as Figures 11 and 12 confirm. This suggests that growth-induced folding can – at least in part – explain cortical thickness variations. At the physical level, these findings are interesting from an instability point of view: While symmetry breaking is not visible in a linear bifurcation analysis, recent studies have shown that it occurs naturally in the non-linear analysis as a consequence of both, the boundary-conditions asymmetry between the external and internal interfaces [87] and the tension-compression asymmetry of the neo-Hookean model [42]. Pattern formation has been extensively studied in bilayered systems with stiff layers on soft substrates [88–91]; yet, understanding morphological instabilities in soft systems remains challenging [92,93]. This is particularly true for instabilities of bilayered systems in the low-stiffness-constrast regime [19,63], which display a wide variety of instability phenomena [41] and have important applications in plant growth [94], seashell growth [95], biofilm growth [96], and embryogenesis [97], where *small variations* in thickness can have *large effects* on the evolution of shape.

## 5 Conclusion

Cortical thickness variations across the human brain play a critical role during neurodevelopment and are characteristic biomarkers for a wide variety of neurological disorders. While recent studies suggest that thickness variations are primarily the result of genetic events, we have demonstrated that geometric and physical events alone could be sufficient to induce cortical patterning and modulate the human cortex. In previous work, we have shown that a growing homogeneous bilayered system undergoes a characteristic symmetry breaking into a spatially heterogeneous system with discrete gyri and sulci [20]. In agreement with magnetic resonance images of infant and adult human brains, our physics-based model explains why gyral regions are universally thicker than sulcal regions. While the observed gyral-to-sulcal thickness ratios can vary in space and time, our theory is generic and equally valid for developmentally early, more folded caudal regions and developmentally late, less folded rostal regions. This suggests that genetic, geometric, and physical factors are tightly interrelated and collectively modulate human brain development. Ultimately, direct measurements of growth patterns – possibly suggested by models such as the current one – are needed to identify the relative roles of genetics, geometry, and physics. Understanding the cause and consequence of cortical thickness variations can provide insight into the development, diagnostics, and treatment of neurological disorders.

## Figures and Tables

**Table 1.** Thickness variations in infant and adult human brains. Gyral thickness, sulcal thickness, and thickness ratio in the temporal, frontal, parietal, and occipital lobes for *n* = 73 infant brains, each scanned at years 0, 1, and 2, and, for comparison, for *n* = 564 healthy brains between years 7 and 64. Gyral thicknesses were significantly larger than sulcal thicknesses (all $p\ll 10^{-10}$) see Figure 8.

	Lobe	Gyral thickness [mm]	Sulcal thickness [mm]	Ratio [−]
Year 0	Temporal	2.236 ± 0.095	1.824 ± 0.068	1.226
	Frontal	2.169 ± 0.093	1.864 ± 0.057	1.164
	Parietal	1.918 ± 0.070	1.837 ± 0.059	1.044
	Occipital	1.794 ± 0.060	1.605 ± 0.117	1.118

Year 1	Temporal	3.372 ± 0.158	2.669 ± 0.114	1.263
	Frontal	3.289 ± 0.155	2.775 ± 0.102	1.185
	Parietal	2.766 ± 0.121	2.583 ± 0.097	1.071
	Occipital	2.246 ± 0.098	2.051 ± 0.092	1.095

Year 2	Temporal	3.329 ± 0.126	2.650 ± 0.108	1.256
	Frontal	3.323 ± 0.105	2.781 ± 0.093	1.195
	Parietal	2.830 ± 0.096	2.526 ± 0.089	1.116
	Occipital	2.278 ± 0.097	2.063 ± 0.089	1.104

Years 7–64	Temporal	3.118 ± 0.300	2.674 ± 0.267	1.166
	Frontal	2.988 ± 0.235	2.410 ± 0.217	1.240
	Parietal	2.761 ± 0.222	2.365 ± 0.229	1.167
	Occipital	2.557 ± 0.218	2.342 ± 0.215	1.092

**Table 2.** Thickness variations in polymer model. Gyral thickness, sulcal thickness, and thickness ratio for varying initial layer thicknesses for regular folding, top, and for period doubling, bottom, at constant prestretch of λ = 2.0, see Figure 9.

	Layer [mm]	Gyral thickness [mm]	Sulcal thickness [mm]	Ratio [−]
Regular	0.300	0.391 ± 0.066	0.307 ± 0.058	1.274
folding	0.600	0.781 ± 0.062	0.524 ± 0.074	1.490
	0.800	1.109 ± 0.131	0.719 ± 0.124	1.543
	1.000	1.506 ± 0.063	0.948 ± 0.094	1.589
	2.000	2.412 ± 0.300	1.487 ± 0.147	1.622

Period	0.450	0.424 ± 0.084	0.332 ± 0.096	1.277
dblg	0.600	0.508 ± 0.071	0.392 ± 0.095	1.295
	1.000	1.246 ± 0.103	0.941 ± 0.195	1.330

**Table 3.** Thickness variations in computational model. Normalized gyral thickness, sulcal thickness, and thickness ratio for increasing growth, see Figure 11.

Growth [−]	Gyral thickness [mm]	Sulcal thickness [mm]	Ratio [−]
1.692	2.519	2.412	1.044
1.735	2.601	2.327	1.118
1.776	2.646	1.850	1.430
1.883	2.835	1.584	1.790
